# While Man Deliberates, Malaria Rules

**DOI:** 10.3201/eid3109.AC3109

**Published:** 2025-09

**Authors:** David O. Freedman

**Affiliations:** University of Alabama at Birmingham, Birmingham, Alabama, USA

**Keywords:** malaria, Dum Romae consulitur, morbos imperat, While Rome Deliberates, the Disease Rules, Giulio Aristide Sartorio, art–science connection, Symbolism, Pontine Marshes, Rome, Italy

**Figure:**
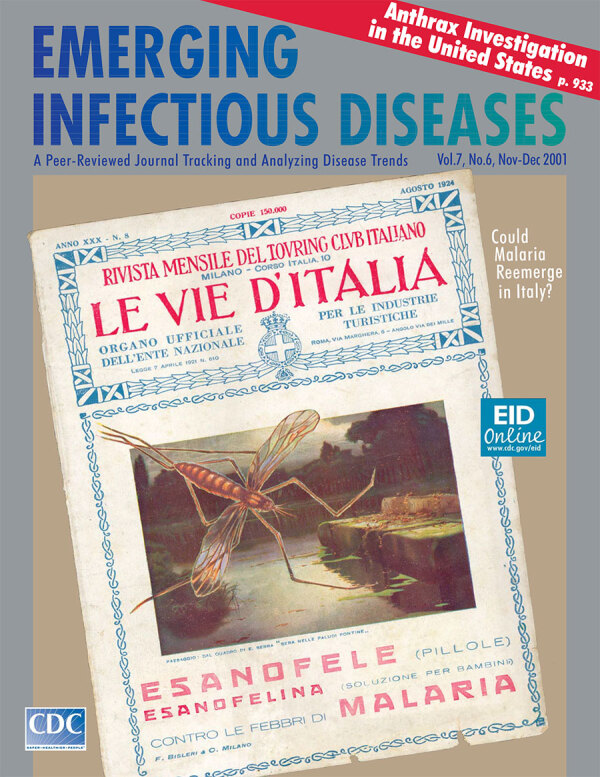
**Giulio Aristide Sartorio (1860–1932), *Malaria***, official title *Dum Romae consulitur, morbos imperat* (1883). Oil on canvas. 125 cm × 223 cm. Museo Nacional de Bellas Artes, Buenos Aires, Argentina. Public domain digital image from Wikipedia Commons.

This month’s cover of *Emerging Infectious Diseases* shows the 1883 painting by Italian artist Giulio Aristide Sartorio (1860–1932) that is commonly known as *Malaria* but officially is titled *Dum Romae consulitur, morbos imperat* (*While Rome Deliberates, the Disease Rules*, an ancient Roman proverb) (Figure). Portraying the devastating human suffering of the disease, the painting depicts life and death in the Pontine Marshes (Agro Pontino), a quadrangular area of now former marshland in the Lazio Region of central Italy, extending along the coast southeast of Rome.

**Figure:**
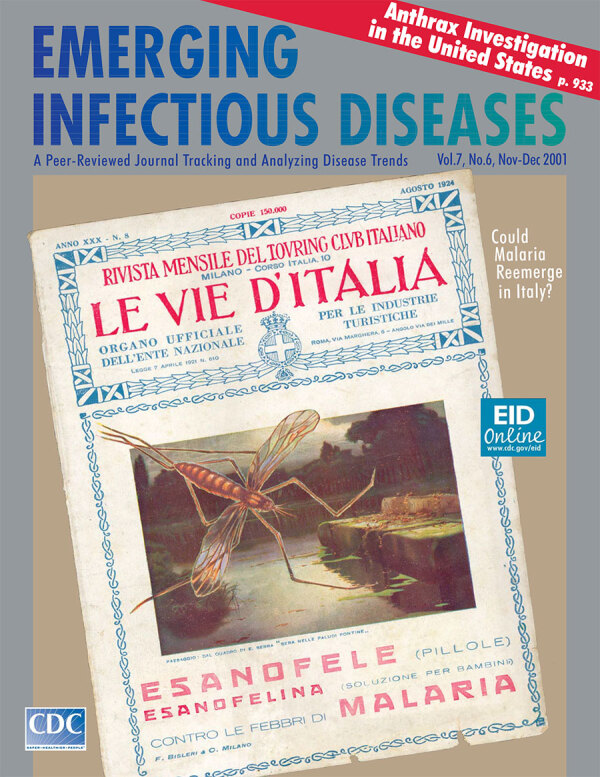
This issue is not the first time the Pontine Marshes of Italy have graced the cover of *Emerging Infectious Diseases*. This 1924 magazine cover from the Touring Club of Milan was featured in the journal’s last bimonthly issue in 2001. Original image provided courtesy of Dr. Guido Sabatinelli.

Sartorio, a notable Italian artist of his time, was a leader of the opposition to the prevailing artistic norms of the Royal Academy, whose proponents he believed were too focused on idealized forms and superficial techniques. Symbolists like Sartorio believed that art could convey deeper, more profound truths by using symbols to represent emotions, ideas, and spiritual experiences. Sartorio produced sophisticated depictions of animals, as well as war paintings, landscapes of the Roman countryside, and the places he visited in Latin America and the East.

Sartorio attended the Institute of Fine Arts beginning in 1876, and commercial success came quickly. In 1883, he debuted *Malaria* at the International Exhibition of Fine Arts in Rome. The dramatic powerful tones and colors and his skill as a daring draftsman, inspired by 17th Century realism, impressed the critics. *Malaria* was purchased in 1885 by a private collector and donated to the Museo Nacional de Bellas Artes in Buenos Aires, Argentina, sometime before 1910. In 1905, Sartorio returned to the theme and exhibited *Marsh Fever*, a very similar malaria-themed painting; its current location is unknown.

*Malaria* portrays the devastating effects of the disease, highlighting the human suffering and environmental conditions of the late 1800s in Symbolist terms. The grim reality of life in the marshy, malaria-ridden Roman campagna shows Sartorio’s skill in capturing social realist themes with a poignant, almost photographic clarity. Social realism draws attention to the real sociopolitical conditions of the working class as a means to critique the power structures behind these conditions. This work signaled his early engagement with contemporary life, exposed the government’s inaction as a public political protest, even as he was mastering the historical and mythological subjects favored by the Academy.

Sartorio treated the subject of a devastated mother kneeling beside her son's corpse with a crude and violent realism. The painting is an ambiguous, still firmly Symbolist, vision of the relationship between death and beauty, reinforced by the scene’s setting in a deserted marsh. The landscape bathed in the light of twilight is a modern reinterpretation of the traditional theme of the pietà, that is, depictions of Mary holding the crucified body of Christ.

Historically, over the centuries since Roman Empire times, the Pontine Marshes have been subject to extensive and expensive periodic land reclamation work. The part of the marsh above sea level needs to be successfully and sustainably drained by channels, so that the highly fertile agricultural land is reclaimed. Whenever the channels are not maintained, the swamp reappears, and mosquito populations explode. Sartorio, therefore, participated in the debate on the cost–benefit of reclamation of the Pontine Marshes by seeking the symbolic reality of humanistic attitudes, which were disappearing from societal norms.

Malaria in the Agro Pontino, present since ancient times, prevented the expansion of Rome to the south. For example, in 1928, during the malaria season, 80% of those having spent 1 night in the marsh became infected. The region had <1,000 inhabitants for a coastal region of >700 km^2^. The settling of the area could provide a new province for Italy and enable settlement that could prevent emigration of 200,000 Italians.

Under Benito Mussolini in the 1930s, the problem was nearly solved by placing dikes and pumping out that portion of the marsh that lay below sea level, but constant maintenance was required. The project reached a peak in 1933 with 124,000 men employed. However, in 1943, just before the Allies landed at the beachhead on the Pontine Marshes for the Battle of Anzio, malaria had returned to the Agro Pontino; quinine and other medicines were in short supply, at the same time infected veterans were returning from the Balkans. In an act interpreted by some as intentional biologic warfare, the Germans flooded the marshes once again. In early 1944, the Battle of Anzio left the marsh in a state of devastation; nearly everything Mussolini had accomplished was reversed. The marshes were full of brackish water, the channels filled in; the mosquitos were flourishing, and malaria was on the rise. Fortunately, the major structures for water control survived, and in a few years, the Agro Pontino was restored. The last of the malaria was conquered in the 1950s with the aid of DDT, and Italy was declared malaria-free by the World Health Organization in 1970. Still, receptive mosquito vectors remain in Italy (and in Greece) and nonsustained autochthonous cases secondary to introduced cases in entering travelers have continued to occur every few years.

In the United States, indigenous transmission of malaria was eliminated in 1951, although ≈2,000 annual malaria cases are imported from malaria-endemic areas. In 2023, nine locally transmitted cases were contracted by US residents who had not recently traveled to endemic areas. Seven cases were recorded in Florida, and 1 each in Texas and Maryland, the result of widespread presence of the *Anopheles* mosquito vector throughout the nonmountainous United States.

The tragedy of malarial childhood death still occurs >400,000 times every year throughout malaria-endemic countries. Since 2022, the World Health Organization has approved 2 separate safe and novel vaccines for infants that are imperfect but can cut those deaths by 30%–50%. Continued need for global support for procurement and implementation programs to achieve vaccination goals remains a priority.
